# Evaluation of Combination Effects of Ethanolic Extract of *Ziziphus mucronata* Willd. subsp. *mucronata* Willd. and Antibiotics against Clinically Important Bacteria

**DOI:** 10.1155/2013/769594

**Published:** 2013-04-24

**Authors:** Olufunmiso Olusola Olajuyigbe, Anthony Jide Afolayan

**Affiliations:** Department of Botany, Phytomedicine Research Centre, University of Fort Hare, Alice 5700, South Africa

## Abstract

A pragmatic approach to the treatment of infectious diseases with multicausal agents and prevention of the development of resistant isolates is the combination of herbal remedies with the first-line antimicrobial agents to which most of them have become resistant. This study evaluated the interactions between the ethanolic bark extract of *Ziziphus mucronata* with known antimicrobial agents *in vitro*. In this study, the results showed that varied zones of inhibitions (ZME—chloramphenicol (17–42 mm), ZME—amoxicillin (17–35 mm), ZME—tetracycline (17–36 mm), ZME—ciprofloxacin (20–41 mm), ZME—nalidixic acid (17–34 mm), and ZME—kanamycin (17–38 mm)) were produced by the antibacterial combinations. At the highest combined concentrations, 12 isolates (ZME—ciprofloxacin) > 10 isolates (ZME—chloramphenicol) = (ZME—kanamycin) > 6 isolates (ZME—amoxicillin) = (ZME—nalidixic acid) and 5 isolates (ZME—tetracycline) were inhibited with zones of inhibition greater than 20 ± 1.0 mm. Although the agar diffusion assay suggested that the interactions between the ethanolic extract of *Z. mucronata* and the antibiotics were both synergistic and additive in nature, the fractional inhibitory concentration indices (FICI) showed that the interactions were synergistic (54.17%), additive (27.78%), indifferent (16.67%), and antagonistic (1.39%). While the fractional inhibitory concentration indices (FICIs) for synergism ranged between 0.00391 and 0.5, that of additivity ranged between 0.516 and 1.0, indifferences ranged between 1.062 and 3.0 and antagonistic interaction was 5.0. The synergistic effects implied that the antibacterial combinations would be more effective and useful in the treatment of multicausal and multidrug-resistant bacteria than a single monotherapy of either antibacterial agent.

## 1. Introduction

Resistance of pathogens to antibiotics is an underappreciated threat to public health in nations around the globe [1]. It is a rapidly growing problem leading to an urgent need for novel antimicrobial agents [2, 3]. While resistant bacteria have become commonplace in healthcare institutions, inadequate empirical therapy resulting in increased mortality rate due to resistant *Pseudomonas aeruginosa, Staphylococcus aureus, Klebsiella pneumoniae, Escherichia coli, Enterobacter* spp., and coagulase-negative staphylococci and enterococci has been reported [4–6]. With this increased incidence of antimicrobial resistance and appearance of new infectious agents, many natural products have been investigated directly for their antimicrobial activity and resistance modifying ability [7, 8]. While the natural products are known to play significant roles in the development of novel drugs and served as leads for the treatment and prevention of diseases [9], plant-derived antimicrobials provide the much needed therapeutics. In phytomedicine research, synergy assessment between medicinal plants and commonly used antibiotics has become a key area of interest because many diseases possess a multicausal agents and complex pathophysiology requiring treatment with well-chosen drug combinations than with a single-drug therapy.

Subsequently, a major strategy that could be employed in the treatment of new emerging infectious diseases and prevention of the development of resistant isolates is the combination of herbal remedies with the first-line antimicrobial agents to which most of them have become resistant. While Kamatou et al. [10] showed that combination of antimicrobial agents had expressed significant interactions, Williamson [11] reported that two or more compounds interact to produce mutual enhancement, amplification, or potentiation of each other's effects when combined. Although these combinations could enhance the efficacy of the other antimicrobial agents and acted as alternative to treating infections caused by multidrug-resistant microorganisms having no effective therapy [12, 13], the pharmacological effects of such mixtures could have resulted from the diverse mechanisms of action resulting from the drug-herbal interactions. Hence, while natural products from plants are considered interesting alternatives for treatment of microbial infections [14, 15], preventing the global increase of undesirable side effects of certain antibiotics and the emergence of previously uncommon infections [16, 17] become imminent with the use of new compounds which are not based on the existing synthetic antimicrobial agents [18].

The genus *Ziziphus* belongs to the Rhamnaceae family. The members of the taxon are drought tolerant and very resistant to heat [19]. *Ziziphus mucronata* Willd. subsp. *mucronata* Willd., known as buffalo thorn, is a small-to-medium-sized tree with a spreading canopy. It is distributed throughout summer rainfall areas of sub-Saharan Africa, extending from South Africa northwards to Ethiopia and Arabia. In ethnomedicine, the pastes of the roots and leaves are used to treat boils, swollen glands, wounds and sores while steam baths from the bark are used to purify and improve skin complexion [20]. Its bark and roots are used for the treatment of rheumatism, gastrointestinal complaints, and snake bites [21]. In East Africa, the roots are used for treating snake bites, gonorrhea, diarrhea, and dysentery [22]. Decoctions of roots and leaves are used to ooze boils and treat sores and glandular swellings [23]. In South Africa, ethnobotanical survey indicated that this plant is used for gastrointestinal disorders including dysentery and diarrhoea [24]. Unlike some members of the *Ziziphus* genus, there is a dearth of scientific reports to indicate the pharmacological activities of this plant. Hence, this study was aimed at evaluating the combination effects of the ethanolic bark extract of *Z. mucronata* and some first-line antibiotics to which microbes have shown resistance against bacteria pathogens that are implicated in clinical infections in order to determine their potential drug-herbal interactions.

## 2. Materials and Methods

### 2.1. Collection of Plant Material

The bark materials of *Ziziphus mucronata* subsp. *mucronata* were collected in August 2010, from the plant growing within the University of Fort Hare campus in Alice, South Africa. The plant was authenticated in the Department of Botany, and a voucher specimen (OLAJ/2010/ZM/01) was prepared and deposited in the Griffin Herbarium of the University.

### 2.2. Extract Preparation

The bark sample was air-dried at room temperature and pulverized using a milling machine. The extract of the bark material was prepared in accordance with the description of Basri and Fan [25]. About 100 g of the pulverized sample was extracted with 500 mL of ethanol for 48 h with shaking (Stuart Scientific Orbital Shaker, UK). The extract was filtered through Whatman no. 1 filter paper and concentrated under reduced pressure at 40°C using a rotary evaporator (Laborota 4000 efficient, Heidolph, Germany). The crude extract collected was allowed to dry at room temperature to a constant weight of 14.2 g. The extract was redissolved in absolute ethanol and made up to the required concentrations for bioassay analysis using sterile deionized distilled water. The reconstituted extract solution was sterilized by filtering through 0.45 *μ*m membrane filter and tested for sterility after membrane filtration by introducing 2 mL of the extract into sterile nutrient broth before being incubated at 37°C for 24 h. A sterile extract was indicated by the absence of turbidity in the broth after the incubation period.

### 2.3. Bacterial Strain

The bacteria used in this study included seven types of culture strains—*Bacillus cereus* ATCC 10702, *Enterococcus faecalis* ATCC 29212, *Escherichia coli* ATCC 8739, *Klebsiella pneumoniae* ATCC 10031, *Proteus vulgaris* ATCC 6830, *Pseudomonas aeruginosa* ATCC 19582, and *Serratia mercescences* ATCC 9986; three environmental strains—*Acinetobacter calcoaceticus anitratus* UP, *Bacillus subtilis* KZN, and *S. flexneri* KZN; and two clinical strains—*S. aureus *OK_2a_ and *S. aureus *OK_2b_. These organisms were obtained from the Department of Biochemistry and Microbiology, University of Fort Hare, Alice, South Africa. The antibacterial assays were carried out using Mueller-Hinton II Agar (Biolab) and broth. The inocula of the test bacteria were prepared using the colony suspension method [26]. Colonies picked from 24 h-old cultures grown on nutrient agar were used to make suspensions of the test organisms in saline solution to give an optical density of approximately 0.1 at 600 nm. The suspension was then diluted 1 : 100 by transferring 0.1 mL of the bacterial suspension to 9.9 mL of sterile nutrient broth before being used.

### 2.4. Antibiotics Used in This Study

Antibiotic powders of amoxicillin, chloramphenicol, ciprofloxacin, tetracycline hydrochloride, kanamycin, and nalidixic acid were used. Stock antibiotic solutions were prepared and dilutions made according to the CLSI (Clinical Laboratory Standardization Institute) method or the manufacturer's recommendations [27, 28].

### 2.5. Antibiotic Susceptibility Testing (Agar Diffusion Method)

Each of the isolates was standardized using colony suspension method. Each strain's suspension was matched with 0.5 McFarland standards to give a resultant concentration of 1.5 × 10^6^ cfu/mL. The antibacterial activity was determined using the well diffusion method according to the modified Kirby-Bauer diffusion technique [29] and the National Committee for Clinical Laboratory Standard [30] by swabbing the Mueller-Hinton agar (MHA) (Oxoid, UK) plates with the resultant saline suspension of each strain. Wells were then bored into the agar medium with heat sterilized 6 mm cork borer. The wells were filled with 100 *μ*L of different concentrations prepared for the methanolic extract alone, antibiotics alone, and their combinations taking care not to allow spillage of the solutions onto the surface of the agar. The plates were allowed to stand for at least 30 min before being incubated at 37°C for 24 h [31]. The determinations were done in duplicate. After 24 h of incubation, the plates were examined for zones of inhibition [32]. The diameter of the zones of inhibition produced by the extract alone, antibiotic alone, and their combinations were measured and interpreted using the CLSI zone diameter interpretative standards [33]. 

### 2.6. Determination of Minimal Inhibitory Concentration (MIC)

The minimum inhibitory concentrations (MICs) for the extract and the antibiotics under study were determined in duplicate by the macrobroth dilution method in Mueller-Hinton broth according to the standard methods of CLSI (Clinical Laboratory Standardization Institute) [27, 28]. To determine the MICs of each antibiotic, 0.0019–500 *μ*g/mL of each of the antibiotics and 0.078–5 mg/mL of the extract were prepared by two-fold serial dilutions in Mueller-Hinton broth. To determine their combinatorial effects, combinations of different concentrations ranging from 0.25x MIC to 8x MIC of each of the antibiotics and the extract were used. The tubes were inoculated with 100 *μ*L of each of the bacterial strains. Blank Mueller-Hinton broth was used as negative control. The bacteria-containing tubes were incubated at 37°C for 24 h. Each combination assay was performed two times. The MIC was taken as the lowest concentration of the extract and the antibiotics that showed no visible growth in the Mueller-Hinton broth after incubation at 37°C for 24 h [34, 35]. 

### 2.7. Checkerboard Assay

The antibacterial effects of combining ethanolic stem bark extract of *Z. mucronata* with antibiotics (chloramphenicol, amoxicillin, tetracycline, ciprofloxacin, nalidixic acid, and kanamycin) were assessed using a checkerboard method [36, 37]. The range of drug concentration used in the checkerboard assay was such that the dilution range encompassed the MIC for each antibiotic used in the analysis. The fractional inhibitory concentration (FIC) was derived from the lowest concentrations of the extract and the antibiotics in combination permitting no visible growth of the test organisms in the Mueller-Hinton broth after incubation at 37°C for 24 h [38]. FIC indices were calculated using the formula: FIC index = (MIC of extract in combination/MIC of extract alone) + (MIC of antibiotics in combination/MIC of antibiotics alone). In agreement with Petersen et al. [37], G. M. Eliopoulos and C. T. Eliopoulos [39], Isenberg [40], and Prinsloo et al. [41], synergy was defined as ∑FIC ≤ 0.5, additivity as 5 < ∑FIC ≤ 1, indifference as 1 < ∑FIC ≤ 4, and antagonism as ∑FIC > 4. 

## 3. Results

In this study, the bacterial isolates exhibited a varied degree of susceptibility to the extract alone, antibiotics alone, and their combinations. At the highest concentration (20 mg/mL), the zones of inhibition for the extract (ZME) ranged between 17 and 27 ± 1.0 mm. At the highest concentration of each of the antibiotics, the zones of inhibition ranged between 15 and 40 ± 1.0 mm for chloramphenicol, 18 and 42 ± 1.0 mm for amoxicillin, 14 and 36 ± 1.0 mm for tetracycline, 17 and 39 ± 1.0 mm for ciprofloxacin, 20 and 29 ± 1.0 mm for nalidixic acid, and 23 and 36 ± 1.0 mm for kanamycin. The agar diffusion assay showed that the isolates exhibited varied degree of concentration-dependent susceptibility to each of the antibacterial combinations. The antibacterial combinations produced zones of inhibition ranging from 17 to 42 ± 1.0 mm for ZME—chloramphenicol, 17–35 ± 1.0 mm for ZME—amoxicillin, 17–36 ± 1.0 mm for ZME—tetracycline, 20–41 ± 1.0 mm for ZME—ciprofloxacin, 17–34 ± 1.0 mm for ZME—nalidixic acid, and 17–38 ± 1. 0 mm for ZME—kanamycin at their respective highest combined concentrations. The inhibition zones from antibacterial combinations were mostly prominent in size than those obtained from either the extract or each of the respective antibiotics used alone. With the exception of *E. coli* ATCC 8739 and *B. cereus* ATCC 10702 being more susceptible to nalidixic acid alone, *S. flexneri* KZN showed the highest susceptibility to the extract, antibiotics, and their combinations. At the highest concentrations combined, 12 isolates (ZME—ciprofloxacin) > 10 isolates (ZME—chloramphenicol) = (ZME—kanamycin) >6  isolates (ZME—amoxicillin) = (ZME—nalidixic acid) and 5 isolates (ZME—tetracycline) were inhibited by the antibacterial combinations with zones of inhibition greater than 20 ± 1.0 mm (Tables 1(a)–1(c)). The agar diffusion assay, therefore, suggested that the interactions between the ethanolic extract of *Z. mucronata* and the antibiotics were both synergistic and additive in nature.

Considering the susceptibility of the individual isolate to the extract and the respective antibiotics, their minimum inhibitory concentrations (MICs) ranged between 0.156 mg/mL and 0.625 mg/mL for the extracts and between 0.0048 *μ*g/mL and 250 *μ*g/mL for all the antibiotics. According to the MIC breakpoints of CLSI [33], the bacteria were classified as being susceptible, intermediate, and resistant based on their susceptibility to each test antibiotic. Susceptible/intermediate/resistant values for each antibiotic—chloramphenicol (≤8/16/≥32 *μ*g/mL/), amoxicillin (≤8/16/≥32 *μ*g/mL), tetracycline (≤4/8/≥16 *μ*g/mL), ciprofloxacin (≤1/2/≥4 *μ*g/mL), nalidixic acid (≤8/16/≥32 *μ*g/mL), and kanamycin (≤16/32/≥64 *μ*g/mL)—were considered as the MIC breakpoints for the antibiotics by the CLSI. Though all the organisms were susceptible to chloramphenicol, tetracycline, and ciprofloxacin, some of them were either resistant or intermediately susceptible to amoxicillin, nalidixic acid, and kanamycin. For the antibiotics, the minimum inhibitory concentrations were in the ranges of 0.977–15.63 *μ*g/mL for chloramphenicol, 0.977–250 *μ*g/mL for amoxicillin, 0.0305–7.813 *μ*g/mL for tetracycline, 0.0048–0.0781 *μ*g/mL for ciprofloxacin, 1.953–250 *μ*g/mL for nalidixic acid, and 1.953–125 *μ*g/mL for kanamycin (Table 2). The fractional inhibitory concentration indices (FICI) showed that interactions between the extract and the antibiotics were synergistic (54.17%), additive (27.78%), indifferent (16.67%), and antagonistic (1.39%). Although the fractional inhibitory concentration of the extract was between 0.0031 and 2.0 and that of the antibiotics was between 0.00391 and 2.0, the fractional inhibitory concentration indices (FICIs) for the antibacterial combinations ranged between 0.00391 and 5.0. While the fractional inhibitory concentration indices (FICIs) for synergism ranged between 0.00391 and 0.5, that of additivity ranged between 0.516 and 1.0, indifferences ranged between 1.062 and 3.0, and antagonistic interaction was 5.0 (Table 3).

## 4. Discussion

The currently observed rapid increase in consumption of herbal remedies worldwide was stirred by several factors including the notion that all herbal products are safe and effective [42, 43]. However, over the past decade, several news-catching episodes in developed communities related life-threatening adverse effects to taking herbal products or traditional medicines [44, 45]. Pak et al. [46] and Saad et al. [47] reported that adulteration, inappropriate formulation, or lack of understanding of plant and drug interactions have led to these adverse reactions that are sometimes life-threatening or lethal to patients. Today, while prescribing the practice of specific class of antibiotics to certain organisms has played critical roles in the development of resistance against that antibiotic [48, 49], combining antibiotics to which microbial resistances have been known globally with medicinal plants, unraveling and understanding antimicrobial resistance would help to minimize the emergence of multidrug-resistant organisms [50]. 

Understanding natural products as a proven template for the development of new scaffolds of drugs [51, 52], the propelling force behind the current trends in phytochemical researches involving herbal-drug interactions is the discovery of new biologically active compounds for medicinal uses. While the success of natural products in drug discovery has been credited to their high chemical density, the effect of evolutionary pressure to create biologically active molecules, and the structural similarity of protein targets across many species [53], the synergy between the ethanolic extract of *Z. mucronata* and the antibiotics demonstrated that there are explorable phytochemicals in the plant that acted synergistically with each of the antibiotics to produce significant antibacterial effects at their supposed target sites. These phytochemicals combining with the antibiotics could have inhibited different stages of some biochemical pathways in the isolates. In both groups of bacteria, the extract could have increased the permeability of the outer membrane barriers by interacting with cell membrane and/or lipopolysaccharide layer to allow the antibiotics to gain access to cytoplasmic targets [54, 55]. While the synergy indicated a broader spectrum of activity and a decreased risk of emergence of resistant strains [56], it could shorten the total duration of therapy and decrease drug related, toxicities by allowing the use of lower doses. Hence, identifying, isolating, and evaluating the promising bioactive phytochemicals in the plant extracts become essential [57].

Consequently, in agreement with previous studies indicating diverse interactions between medicinal plants and different antibiotics [8, 58–60], this study showed that the combination of ethanolic extract of *Z. mucronata* with the antibiotics was more synergistic than being indifferent or antagonistic. The antibacterial combinations resulted in synergy that strongly inhibited the growth of the bacterial isolates. Although the indiscriminate use of antimicrobial agents in the treatment of bacterial infections has led to the emergence of resistant strains and a great loss of clinical efficacy of previously effective first-line antimicrobials resulting in the shifting of antimicrobial treatment regimen to second-line or third-line antimicrobial agents that are often more expensive with many side effects [61], the synergistic interaction of the extract of *Z. mucronata* and the antibiotics could be a powerful tool in preventing or suppressing the emergence of resistant strains, decreasing dose-related toxicity, attaining a broad spectrum of activity [62], and selecting appropriate antimicrobial therapy [63, 64]. The synergistic effects of these combinations would, therefore, be useful in the treatment of multicausal and multidrug-resistant bacteria [65–67].

## 5. Conclusions

Resistance to antibiotics is a ubiquitous and relentless clinical problem compounded by a dearth of new therapeutic agents. The retreat of the pharmaceutical industries from research and development of new antibiotic has exacerbated the challenge of widespread resistance and signals a critical need for innovation. Although antimicrobial combinations are commonly used in medicine to broaden antimicrobial spectrum and generate synergism, it should be promoted and encouraged as a strategy for reducing the emergence of antibiotic-resistant strains. This study showed that antibacterial combination of extract of *Z. mucronata* with the different antibiotics was more of synergy and would be effective in the treatment of microbial infections in which multidrug-resistant bacteria are involved. The active compounds in *Ziziphus mucronata*, if isolated, may be used as a therapeutic drug candidate for controlling microbial infections. Further research involving interaction of the isolated pure compounds and antibiotics as well as *in vitro* determination of mechanisms of action would be further investigated in our laboratory.

## Figures and Tables

**Table:** (a)

				Zones of inhibition (±1.0 mm) produced by the extract, antibiotics, and their combinations *in vitro *											
	ZME			Chl			ZME + Chl			Amx			ZME + Amx		
	5	10	20	62.5	125	250	5 + 62.6	10 + 125	20 + 250	62.5	125	250	5 + 62.5	10 + 125	20 + 250
	(mg/mL)			(*µ*g/mL)			(mg/mL + *µ*g/mL)			(*µ*g/mL)			(mg/mL + *µ*g/mL)		
A	17	18	20	23	27	30	20	25	28	20	23	26	0	14	17
B	17	18	20	20	25	30	22	26	29	14	16	21	0	13	16
C	17	18	20	23	27	30	23	25	27	16	19	21	0	13	16
D	15	16	18	21	27	30	23	25	29	15	18	20	13	15	18
E	14	15	17	22	25	29	22	26	28	30	32	34	28	31	34
F	16	17	19	22	26	30	20	26	29	0	15	18	13	16	18
G	16	17	18	22	27	31	19	22	25	13	16	18	0	12	16
H	15	17	19	23	25	27	20	23	26	28	30	32	28	30	33
I	14	15	18	0	0	15	13	15	17	30	34	37	28	31	34
J	15	17	18	23	25	27	0	15	17	30	33	35	27	31	33
K	15	16	18	20	24	27	23	25	28	27	31	35	25	28	30
L	23	25	27	33	37	40	38	40	42	31	36	42	29	33	35

**Table:** (b)

				Zones of inhibition (±1.0 mm) produced by the extract, antibiotics, and their combinations *in vitro *											
		ZME		Tet			ZME + Tet			Cip			ZME + Cip		
	5	10	20	62.5	125	250	5 + 62.6	10 + 125	20 + 250	1.25	2.5	5	5 + 1.25	10 + 2.5	20 + 5
	(mg/mL)			(*µ*g/mL)			(mg/mL + *µ*g/mL)			(*µ*g/mL)			(mg/mL + *µ*g/mL)		
A	17	18	20	0	15	18	0	15	17	27	30	35	20	22	25
B	17	18	20	0	0	14	0	15	16	18	21	24	18	20	22
C	17	18	20	0	0	15	13	15	17	13	15	19	21	24	26
D	15	16	18	0	13	16	0	14	16	19	20	22	18	22	25
E	14	15	17	25	28	32	20	22	25	23	26	32	15	17	20
F	16	17	19	0	0	15	13	15	17	13	15	17	20	23	27
G	16	17	18	0	15	17	13	15	19	18	21	24	20	23	25
H	15	17	19	25	27	30	22	25	27	17	20	23	26	29	31
I	14	15	18	0	13	16	0	15	17	17	21	24	15	18	22
J	15	17	18	25	27	30	15	18	20	13	15	17	16	19	23
K	15	16	18	25	28	31	20	23	25	25	27	30	15	16	20
L	23	25	27	29	32	36	30	33	36	30	35	39	33	37	41

**Table:** (c)

				Zones of inhibition produced by the extract, antibiotics, and their combinations *in vitro *											
	ZME			Nal			ZME + Nal			Kan			ZME + Kan		
	5	10	20	62.5	125	250	5 + 62.6	10 + 125	20 + 250	62.5	125	250	5 + 62.5	10 + 125	20 + 250
	(mg/mL)			(*µ*g/mL)			(mg/mL + *µ*g/mL)			(*µ*g/mL)			(mg/mL + *µ*g/mL)		
A	17	18	20	21	26	29	15	16	18	26	29	32	24	26	29
B	17	18	20	21	25	29	13	15	17	24	25	28	23	24	28
C	17	18	20	19	22	26	15	17	20	25	27	29	23	25	28
D	15	16	18	20	23	25	13	15	17	24	27	30	24	26	28
E	14	15	17	16	20	25	23	24	26	19	21	23	17	19	22
F	16	17	19	18	21	25	14	16	17	25	26	28	22	24	27
G	16	17	18	20	22	26	14	16	18	25	26	30	23	25	28
H	15	17	19	17	20	23	20	21	25	20	23	25	18	20	23
I	14	15	18	17	21	25	13	16	18	21	24	27	21	23	25
J	15	17	18	15	17	20	25	28	30	19	20	23	13	15	19
K	15	16	18	23	26	27	21	24	26	22	24	25	0	14	17
L	23	25	27	22	24	28	25	28	34	30	32	36	32	25	38

Key: A: *E. coli* ATCC 8739; B: *B. cereus* ATCC 10702; C: *B. subtilis *KZN; D: *P. aeruginosa* ATCC 19582; E: *S. marcescens* ATCC 9986; F: *A. calcoaceticus anitratus* UP; G: *K. pneumoniae* ATCC 10031; H: *P. vulgaris* ATCC 6830; I: *E. faecalis* ATCC 29212; J: *S. aureus* OK_2a_; K: *S. aureus* OK_2b_; L: *S. flexneri *KZN.

**Table 2:** Susceptibility of the bacterial isolates to *Ziziphus mucronata *extract and the antibiotic.

	Minimum Inhibitory concentrations of extracts and the different antibiotics used in combination						
	ZME	Chloramphenicol	Amoxicillin	Tetracycline	Ciprofloxacin	Nalidixic acid	Kanamycin
	(mg/mL)	(*µ*g/mL)					
*E. coli* ATCC 8739	0.156	15.63 (I)	15.63 (S)	0.977 (S)	0.0048 (S)	3.906 (S)	31.25 (I)
*B. cereus* ATCC 10702	0.156	3.91 (S)	7.813 (S)	0.0305 (S)	0.0781 (S)	15.63 (I)	125 (R)
*B. subtilis *KZN	0.313	3.91 (S)	62.5 (R)	0.977 (S)	0.0195 (S)	7.813 (S)	3.906 (S)
*P. aeruginosa* ATCC 19582	0.313	3.91 (S)	3.906 (S)	0.488 (S)	0.156 (S)	7.813 (S)	31.25 (I)
*S. marcescens* ATCC 9986	0.625	0.98 (S)	31.25 (I)	1.563 (S)	0.0781 (S)	1.953 (S)	1.953 (S)
*A. calcoaceticus anitratus *	0.313	7.81 (S)	250 (R)	0.49 (S)	0.0195 (S)	31.25 (S)	15.63 (S)
*K. pneumoniae* ATCC 10031	0.156	1.95 (S)	0.977 (S)	0.488 (S)	0.039 (S)	3.906 (S)	15.63 (S)
*P. vulgaris* ATCC 6830	0.313	7.81 (S)	250 (R)	7.813 (I)	0.0195 (S)	1.953 (S)	31.25 (I)
*E. faecalis* ATCC 29212	0.313	1.95 (S)	3.906 (S)	3.906 (S)	0.313 (S)	31.25 (I)	125.00 (R)
*S. aureus* OK_2a_	0.156	7.81 (S)	125 (R)	0.977 (S)	0.0781 (S)	31.25 (I)	7.813 (S)
*S. aureus* OK_2b_	0.313	7.81 (S)	7.813 (S)	0.197 (S)	0.0195 (S)	62.50 (R)	31.25 (I)
*S. flexneri *KZN	0.313	7.813 (S)	250 (R)	0.197 (S)	0.039 (S)	250 (R)	15.625 (S)

**Table 3:** Effects of combining ethanol extract of *Ziziphus mucronata* subsp.* mucronata* with antimicrobial agents against selected bacterial strains.

		Effects of the combined ethanol extract and different antibacterial agents on the tested bacterial isolates											
		A	B	C	D	E	F	G	H	I	J	K	L
	FICE	0.25	0.25	0.25	0.25	0.125	2	1	0.125	0.25	0.125	0.25	0.125
Chloramphenicol	FICAs	0.125	0.125	0.5	0.25	0.25	1	2	0.25	0.125	0.25	0.25	0.5
	FICIs	0.375	0.375	0.75	0.5	0.375	3	3	0.375	0.375	0.375	0.5	0.625
	Rem	Syn	Syn	Add	Syn	Syn	Ind	Ind	Syn	Syn	Syn	Syn	Add

	FICE	0.25	1	0.25	0.125	0.125	0.125	0.5	0.125	0.031	0.25	0.031	0.031
Amoxicillin	FICAs	0.25	1	0.125	0.125	0.125	0.003906	0.25	0.25	0.25	0.25	0.125	0.5
	FICIs	0.5	2	0.375	0.25	0.25	0.129	0.75	0.375	0.281	0.5	0.156	0.531
	Rem	Syn	Ind	Syn	Syn	Syn	Syn	Add	Syn	Syn	Syn	Syn	Add

	FICE	0.125	0.062	0.125	0.062	0.25	0.25	1	0.125	0.5	0.25	0.062	0.125
Tetracycline	FICAs	0.5	0.125	0.25	1	0.25	0.016	1	0.25	0.25	0.5	0.125	0.5
	FICIs	0.625	0.187	0.375	1.062	0.75	0.344	2	0.375	0.75	0.75	0.187	0.625
	Rem	Add	Syn	Syn	Ind	Add	Syn	Ind	Syn	Add	Add	Syn	Add

	FICE	0.25	0.25	0.125	0.25	1	1	0.5	0.062	0.125	0.25	0.125	0.25
Ciprofloxacin	FICAs	0.125	0.25	0.125	0.25	1	1	0.125	0.031	0.125	0.25	0.25	0.125
	FICIs	0.375	0.5	0.25	0.5	2	2	0.625	0.094	0.25	0.5	0.375	0.375
	Rem	Syn	Syn	Syn	Syn	Ind	Ind	Add	Syn	Syn	Syn	Syn	Syn

	FICE	0.25	2	1	0.2496	0.125	0.125	0.5	0.25	0.125	0.5	0.25	1
Nalidixic acid	FICAs	0.125	1	2	0.5	2	0.5	0.5	0.5	0.5	0.5	0.5	4
	FICIs	0.375	3	3	0.75	2.125	0.625	1	0.75	0.625	1	0.75	5
	Rem	Syn	Ind	Ind	Add	Ind	Add	Add	Add	Add	Add	Add	Ant

	FICE	0.25	0.25	0.5	0.5	0.125	0.125	0.25	0.125	0.5	1	0.25	0.062
Kanamycin	FICAs	0.125	0.063	0.25	0.125	0.5	0.0625	0.125	0.25	0.01563	0.5	0.125	0.031
	FICIs	0.375	0.313	0.75	0.625	0.625	0.188	0.375	0.375	0.516	1.5	0.375	0.094
	Rem	Syn	Syn	Add	Add	Add	Syn	Syn	Syn	Add	Ind	Syn	Syn

Key: A: *E. coli* ATCC 8739; B: *B. cereus* ATCC 10702; C: *B. subtilis *KZN; D: *P. aeruginosa* ATCC 19582; E: *S. marcescens* ATCC 9986; F: *A. calcoaceticus anitratus* UP; G: *K. pneumoniae* ATCC 10031; H: *P. vulgaris* ATCC 6830; I: *E. faecalis* ATCC 29212; J: *S. aureus* OK_2a_, K: *S. aureus* OK_2b_, L: *S. flexneri *KZN; Rem: remarks; Syn: synergy; Ant: antagonisms; add: additivity; FICE: FIC of extract; FICAs: FIC of antibiotics; FICIs: FIC indices.
